# Mean platelet volume is associated with periodontitis: a cross-sectional study

**DOI:** 10.1186/s12903-024-04223-8

**Published:** 2024-04-16

**Authors:** Chenying Zhou, Ye Liu, Jingjing Bai, Yi Luo, Jukun Song, Ping Feng

**Affiliations:** 1 School of Stomatology, Zunyi Medical University, Zunyi, Guizhou China; 2 The Affiliated Stomatological Hospital and Stomatology of Guizhou Medical University, Guizhou Medical University, Guiyang, China; 3 Guiyang Hospital of Stomatology, Guiyang, Guizhou China

**Keywords:** Cross-sectional study, Mean platelet volume, NHANES, Periodontal health

## Abstract

**Background:**

It is uncertain if mean platelet volume and periodontitis are related. The objective of this study was to examine the association between levels of mean platelet volume and moderate/severe periodontitis in adult persons who inhabit the U.S.

**Methods:**

We screened 6,809 people from the National Health and Nutrition Examination Survey (NHANES 2009–2012). Mean platelet volume was measured in the Mobile Examination Centers (MECs) using the Beckman Coulter analyzer. The category of periodontitis was defined by the CDC/AAP using clinical periodontal parameters. Multiple logistic regression models were employed to examine the distribution for covariate differences across the various independent groups. Four models were employed to examine the relationship between mean platelet volume level and periodontitis. Smoothed curve fitting was utilized to confirm the linearity of the relationships. To determine the impact of factors on the connection between MPV and periodontitis, subgroup analysis and interaction testing were utilized.

**Results:**

Results from the multiple logistic regression analysis indicate a significant association between moderate/severe periodontitis and the mean platelet level, even after considering any potential confounding variables (OR = 1.090, 95% CI: 1.019–1.166, P-value = 0.01211). Additionally, those in the upper tertile of mean platelet volume levels had a 21.6% higher probability of developing periodontitis when compared with those in the least tertile of mean platelet levels (OR = 1.216, 95% CI:1.052–1.406, P-value = 0.00816). Moreover, it showed a positive correlation between mean platelet volume (MPV) and moderate/severe periodontitis. Subgroup analyses indicated a positive association between the level of mean platelet volume and moderate/severe periodontitis among individuals who were under 60 years of age, had low income, were obese, never smoked, were heavy drinkers, had hypertension, and had no cardiovascular disease (*p* < 0.05). However, none of the subgroups exhibited significant interactions (p for interaction > 0.05).

**Conclusion:**

A correlation has been found between mean platelet volume levels and periodontal disease in individuals residing in the United States.

## Introduction

Periodontitis is the sixth most prevalent disease globally [1]. Periodontitis affects a significant proportion of adults aged 30 and older in the United States, with an estimated frequency of approximately 46% [2]. Periodontitis is a multifactorial inflammatory illness produced by particular microorganisms that damages the supporting tissues of the teeth [3]. For these microbes to survive, replicate or multiply, they induce an inflammatory response in the host tissue. To neutralize this, the body also triggers the host response to fight off these pathogens [4]. Comparing patients with periodontitis to healthy controls, cross-sectional studies have revealed that the former have higher levels of systemic inflammatory markers, such as lymphocytes, leukocytes, erythrocyte distribution width, mean corpuscular volume [4], and C-reactive protein [5]. Periodontal inflammation may worsen systemic diseases by inducing pathological changes through the production of specific molecules by leukocytes, particularly neutrophils, that are directly responsible for the inflammatory response [6]. The immunomodulatory function of lymphocytes also plays a critical role in periodontal inflammation [7]. Several blood inflammatory markers such as neutrophil-to-lymphocyte ratio (NLR), lymphocyte-to-monocyte ratio (LMR), are considered emerging biomarkers of inflammation in blood counts, to be used as prognostic indicators in periodontitis [8].

However, platelets, another major component of blood, have received little attention in relation to periodontal inflammation. In vitro and animal studies have shown that Porphyromonas gingivalis [9], a periodontal pathogen, as well as other plaque bacteria such as Streptococcus haematobium, induce platelet activation and aggregation [10]. Upon encountering pathogens, platelets release a wide range of immunomodulatory cytokines, chemokines, and other mediators [11]. They modulate neutrophil activity, promoting their aggregation to sites of tissue damage and direct killing of infected cells, and platelet-inflammatory cell interactions may mediate pro-inflammatory outcomes and facilitate infection limitation [12].

Mean platelet volume (MPV) is a reliable indicator of platelet function and activation and is often considered a marker of inflammation in various chronic diseases [13]. For example, MPV has been shown to be a biomarker for platelet activation [14], risk stratification and diagnosis in patients with coronary artery disease [15] and a biomarker for the identification of microvascular complications in diabetes [16]. Additionally, patients with rheumatoid arthritis [17] and non-alcoholic fatty liver disease [18] have higher levels of MPV. However, few studies have investigated MPV levels in periodontology, despite the association between periodontitis and platelet activation [15]. In the study by Roarigo et al., slightly elevated MPV levels were observed in patients with periodontitis [19]. However, the patients included in the study were adolescents with mild periodontitis. Other studies found elevated MPV values in the periodontitis group compared to healthy controls, but their sample population was only 57 [6].

Mean platelet counts, which are routinely reported as part of complete blood count (CBC) analyses, are simple and inexpensive but often receive little attention. We hypothesize that chronic inflammation may be the common pathophysiological link between elevated MPV levels and periodontitis. To investigate this, we evaluated data from the National Health and Nutrition Examination Survey (NHANES, 2009–2012) to discover any association between MPV and periodontitis. This association may hold great promise for the prevention of periodontitis.

## Methods

### Sample and data sources

The National Health and Nutrition Examination Survey (NHANES) is a national, representative, and publicly available database designed to collect data on the potential causes that may affect the health and nutritional status of Americans [20]. NHANES is a major program of the National Center for Health Statistics (NCHS), which is part of the Centers for Disease Control and Prevention (CDC) and is responsible for producing vital statistics and health statistics for the United States. The NHANES protocols were approved by the NCHS Ethics Review Board and all participants were informed of the need to provide informed consent. Ethical approval for the study [21] and data release and access policy of NHANES [22] can be found at NHANES website.

In accordance with the objective of this cross-sectional investigation, we collected data from NHANES 2009–2010 and 2011–2012, using the specified inclusion criteria: Participants aged 30 years or above who received a comprehensive Full Mouth Periodontal Examination (FMPE) and had available data on mean platelet volume (MPV).

### Exposure variable

Mean Platelet Volume was measured using the ethylenediaminetetraacetic acid-mixed blood sample from the participants as part of a complete blood count with the Coulter HMX Hematology Analyzer. The normal range for MPV was 9.6–12.6 f L [23].

### Outcome variable

Every individual in the NHANES 2009–2010, 2011–2012 discovery dataset received a comprehensive evaluation of their whole mouth, including the four quadrants, to determine the presence of either no/mild or moderate/severe periodontitis. Dentists who had received training and calibration inspected each subject. In accordance with the FMPE protocol, the periodontal examination included assessing the clinical attachment loss (CAL) and probing pocket depth (PPD) at six specific places on each tooth, except the third molars. To assess the periodontal status, a maximum of 168 locations and 28 teeth per patient were examined.

We employed the CDC/AAP (Centers for Disease Control and Prevention and American Academy of Periodontology) case definitions for the classification of periodontal disease [24]. “No or mild periodontitis” is the term used to describe the absence of moderate or severe periodontitis. Moderate/severe periodontitis is defined by the presence of clinical attachment loss (CAL) of 4 mm or more (not on the same tooth) or 2 or more interproximal areas, together with probing pocket depth (PPD) more than 5 mm. The participants were categorized into two groups according to the severity of their periodontitis: no/mild and moderate/severe [25].

### Covariates

We collected covariates that may influence the relationship between mean platelet volume and periodontitis, mainly including sociodemographic, lifestyle, laboratory test indicators, and chronic disease variables. Specifically, continuous variables included age, hemoglobin, hematocrit, white blood cell count, neutrophils, lymphocytes, mean corpuscular volume, mean corpuscular hemoglobin, mean corpuscular hemoglobin concentration, red blood cell distribution width, total platelet count [8, 26]. These blood parameters are calculated from blood samples taken in the Mobile Examination Centre (MEC) using the Beckman Coulter MAXM instrument. The categorical covariates include gender(male or female); marital status (married/living with a partner, widowed/divorced/separated, never married); race(Mexican American, non-Hispanic White, non-Hispanic Black, other races); education level (high and above or below high school); poverty income ratio (PIR) (low (< 1.3), middle (1.3–3.5), high (> 3.5)) [27]; body mass index (BMI) (not overweight (< 25 kg/m^2^), overweight (25–30 kg/m^2^), obesity (≥ 30 kg/m^2^)) [28]; hypertension(yes or no); diabetes(yes or no); cardiovascular disease(yes or no) [29]. Smoking habit was classified as never, previous and current smokers, based on their responses to questionnaires on whether they had smoked at least 100 cigarettes in their lifetime and whether they were current smokers [30]. The National Institute on Alcohol Abuse and Alcoholism (NIAAA) has established definitions for alcohol consumption, categorized as none, moderate (1–2 drinks per day for men, or 1 drink per day for women), and heavy (3–4 drinks per day for men or 2–3 drinks per day for women) [31].

### Statistical analysis

Descriptive data statistics are used for concisely describe the basic characteristics of the study population. Categorical variables are displayed as frequencies or percentages, while continuous variables that adhere to a normal distribution are described by their means and standard deviations.

The statistical analysis was separated into three primary stages to establish the correlation between the severity of periodontitis and the mean platelet volume of the study participants. Firstly, the baseline data distribution for the different degrees of periodontitis in the participants of this investigation was presented. To demonstrate the distinctions among the different categories of periodontal severity, we used a one-way ANOVA for normally distributed data, a chi-square test for categorical variables, and a Kruskal-Wallis test for non-normally distributed data. During the second phase, we utilized logistic regression modeling to predict marginal proportions and calculate adjusted risk ratios in order to investigate the connection of MPV and moderate/severe periodontal disease. We constructed four statistical models: model I, without accounting for confounding variables; model II, the adjustment is made just for age, gender, race, education level, marital status, and PIR; model III, adjusting only for age, gender, race, education level, marital status, PIR, BMI, smoking habits, drinking habits, high blood pressure, cardiovascular disease, and diabetes; Model IV, accounting for all variables, age, gender, race, education level, marital status, PIR, BMI, smoking habit, drinking habit, hypertension, cardiovascular disease, diabetes, hemoglobin, mean corpuscular hemoglobin, hematocrit, neutrophils, mean corpuscular volume, white blood cell count, lymphocytes, red blood cell distribution width, mean corpuscular hemoglobin concentration, total platelet count. In addition, smoothed curve fitting was used to validate linear relationships. Finally, to assess the relationship between MPV and periodontitis across various demographic characteristics, while maintaining consistency, subgroup analyses and interaction tests were conducted. Analyses were stratified by age, gender, race, education level, marital status, PIR, BMI, smoking habits, drinking habits, and the existence of chronic illnesses (such as hypertension, cardiovascular disease, and diabetes mellitus).

The statistical program R (R Foundation)(http://www.r-project.org) and Empower Stats( http://www.empowerstats.net/cn/index.php) were used to conduct all analyses. Statistical significance was defined as a P-value less than 0.05.

## Results

### Examination samples’ basic characteristics

**Fig. 1:**
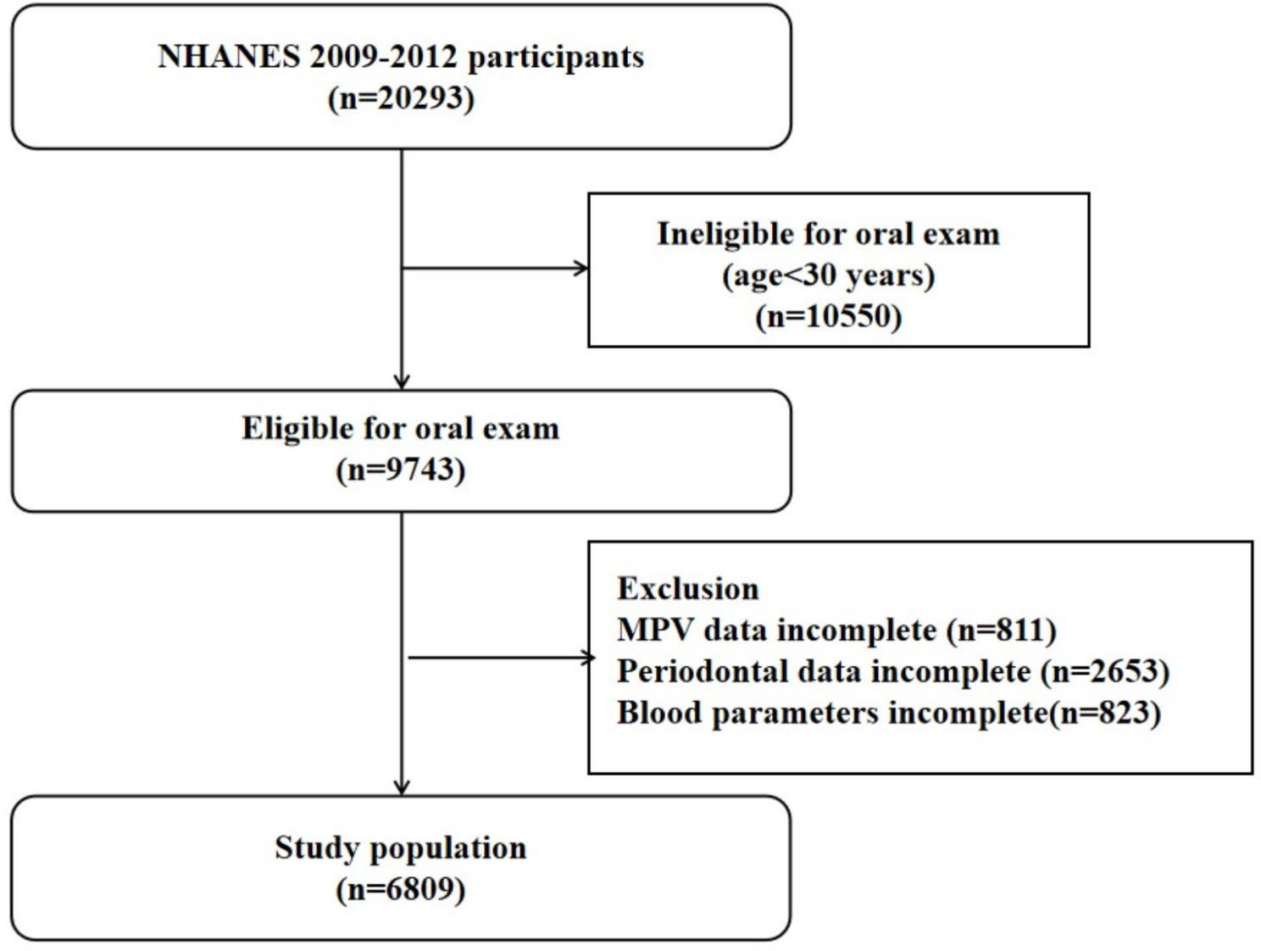
Flowchart of sample selection. Study population selection (*N* = 6809). MPV, Mean Platelet Volume; NHANES, National Health and Nutrition Examination Survey

Figure 1 shows that a total of 6809 individuals from the NHANES dataset were included in this analysis. Table 1 presents the fundamental characteristics for the participants involved in the research. The study population consisted of 6809 subjects with a mean age of 52.03 ± 14.26 years, including 3389 males and 3420 females. The participants were classified into two groups according to their periodontal condition: a group with no/mild periodontitis (*n* = 3504) and a group with moderate/severe periodontitis (*n* = 3305). When comparing the group of individuals with no/mild periodontitis to the group of individuals with moderate/severe periodontitis, the latter group was found to be older (56.85 ± 13.61 vs. 47.48 ± 13.35), to be male (59.09% vs. 40.98%), and to be smokers, including previous smokers (27.90% vs. 22.43%) and current smokers (24.78% vs. 13.56%). In addition, patients with no/mild periodontitis appeared to be more likely to be married/living with a partner (67.24% vs. 61.79%), non-Hispanic white (50.00% vs. 36.46%), high school and above education level (83.02% vs. 66.38%), high income (39.44% vs. 22.24%), and heavy drinkers (37.90% vs. 37.85%) had higher proportions. In terms of comorbidities, the prevalence of hypertension (60.45% vs. 42.78%), diabetes (17.67% vs. 8.56%), and CVD (12.44% vs. 4.62%) seemed to be higher in those suffering from moderate/severe periodontitis. Furthermore, individuals who had moderate/severe periodontitis had elevated levels of hemoglobin (14.07 ± 1.51), hematocrit, (41.26 ± 4.28), white blood cell count (7.16 ± 2.91), neutrophils (4.27 ± 2.41), mean corpuscular volume (90.17 ± 5.96), mean erythrocyte hemoglobin ( 30.75 ± 2.40), and red blood cell distribution width (13.10 ± 1.36) in comparison to those with no/mild periodontitis, whereas the mean corpuscular hemoglobin concentration (34.09 ± 1.01) and total platelet count (235.25 ± 64.70) were lower.

**Table 1:** Participant characteristics in the NHANES 2009–2012

Characteristics	Total (*N* = 6809)	No/Mild (*N* = 3504)	Moderate/Severe (*N* = 3305)	P-value
**Age(years)**	52.03 ± 14.26	47.48 ± 13.35	56.85 ± 13.61	< 0.001
**Gender**				< 0.001
Male	3389 (49.77%)	1436 (40.98%)	1953 (59.09%)	
Female	3420 (50.23%)	2068 (59.02%)	1352 (40.91%)	
**Marital status**				< 0.001
Married/Living with a partner	4398 (64.59%)	2356 (67.24%)	2042 (61.79%)	
Widowed/divorced/separated	1629 (23.92%)	699 (19.95%)	930 (28.14%)	
Never married	777 (11.41%)	448 (12.79%)	329 (9.95%)	
Missing	5 (0.07%)	1 (0.03%)	4 (0.12%)	
**Race**				< 0.001
Mexican American	1006 (14.77%)	427 (12.19%)	579 (17.52%)	
Non-Hispanic White	2957 (43.43%)	1752 (50.00%)	1205 (36.46%)	
Non-Hispanic Black	1407 (20.66%)	594 (16.95%)	813 (24.60%)	
Other	1439 (21.13%)	731 (20.86%)	708 (21.42%)	
**Education level**				< 0.001
High and above	5103 (74.94%)	2909 (83.02%)	2194 (66.38%)	
Below high school	1695 (24.89%)	591 (16.87%)	1104 (33.40%)	
Missing	11 (0.16%)	4 (0.11%)	7 (0.21%)	
**PIR**				< 0.001
Middle income	2239 (32.88%)	1098 (31.34%)	1141 (34.52%)	
Low income	1869 (27.45%)	759 (21.66%)	1110 (33.59%)	
High income	2117 (31.09%)	1382 (39.44%)	735 (22.24%)	
Missing	584 (8.58%)	265 (7.56%)	319 (9.65%)	
**BMI**				0.866
Not overweight	1807 (26.54%)	933 (26.63%)	874 (26.44%)	
Overweight	2363 (34.70%)	1211 (34.56%)	1152 (34.86%)	
Obesity	2595 (38.11%)	1340 (38.24%)	1255 (37.97%)	
Missing	44 (0.65%)	20 (0.57%)	24 (0.73%)	
**Smoking habit**				< 0.001
Never	3804 (55.87%)	2243 (64.01%)	1561 (47.23%)	
Previous	1708 (25.08%)	786 (22.43%)	922 (27.90%)	
Current	1294 (19.00%)	475 (13.56%)	819 (24.78%)	
Missing	3 (0.04%)	0 (0.00%)	3 (0.09%)	
**Drinking habit**				< 0.001
Never	832 (12.22%)	375 (10.70%)	457 (13.83%)	
Moderate	2018 (29.64%)	1154 (32.93%)	864 (26.14%)	
Heavy	2579 (37.88%)	1328 (37.90%)	1251 (37.85%)	
Missing	1380 (20.27%)	647 (18.46%)	733 (22.18%)	
**Hypertension**				< 0.001
No	3177 (46.66%)	1916 (54.68%)	1261 (38.15%)	
Yes	3497 (51.36%)	1499 (42.78%)	1998 (60.45%)	
Missing	135 (1.98%)	89 (2.54%)	46 (1.39%)	
**Diabetes**				< 0.001
No	5784 (84.95%)	3136 (89.50%)	2648 (80.12%)	
Yes	884 (12.98%)	300 (8.56%)	584 (17.67%)	
Missing	141 (2.07%)	68 (1.94%)	73 (2.21%)	
**CVD**				< 0.001
No	6227 (91.45%)	3341 (95.35%)	2886 (87.32%)	
Yes	573 (8.42%)	162 (4.62%)	411 (12.44%)	
Missing	9 (0.13%)	1 (0.03%)	8 (0.24%)	
**Hb(g/dL)**	14.01 ± 1.49	13.94 ± 1.46	14.07 ± 1.51	< 0.001
**Hct (p g)**	40.97 ± 4.23	40.70 ± 4.17	41.26 ± 4.28	< 0.001
**WBC (10** ^**3**^ **/µ L)**	7.02 ± 2.48	6.88 ± 1.97	7.16 ± 2.91	< 0.001
**NEUT (10** ^**3**^ **/µ L)**	4.17 ± 2.02	4.08 ± 1.56	4.27 ± 2.41	< 0.001
**LYMPH (10** ^**3**^ **/µ L)**	2.09 ± 0.79	2.07 ± 0.67	2.10 ± 0.90	0.514
**MCV (f L)**	89.69 ± 5.77	89.24 ± 5.55	90.17 ± 5.96	< 0.001
**MCH (p g)**	30.67 ± 2.34	30.59 ± 2.29	30.75 ± 2.40	0.003
**MCHC(g/dL)**	34.17 ± 1.00	34.25 ± 1.00	34.09 ± 1.01	< 0.001
**RDW (%)**	12.99 ± 1.32	12.89 ± 1.28	13.10 ± 1.36	< 0.001
**TPC (10** ^**3**^ **/µ L)**	239.00 ± 62.81	242.53 ± 60.78	235.25 ± 64.70	< 0.001

Mean ± standard deviation for the continuous variables. N (percentage) for the categorical variables

PIR, ratio of family income to poverty; BMI, body mass index; CVD, cardiovascular disease; Hb, hemoglobin; Hct, hematocrit; MCH, mean corpuscular hemoglobin; MCHC, mean corpuscular hemoglobin concentration; RDW, red blood cell distribution width; WBC, white blood cell count; NEUT, neutrophils; MCV, mean corpuscular volume; LYMPH, lymphocytes; TPC, total platelet count

### Mean platelet volume and periodontitis

Table 2 displays the correlation between MPV levels and periodontitis. Model I suggests that a rise in MPV level is associated with higher probabilities of moderate/severe periodontitis, with an OR (odds ratio) = 1.068 (95% CI: 1.015–1.123) and *p* = 0.01193. Comparable results were found in crude-adjusted model II: OR = 1.072 (95% CI: 1.012–1.137), *p* = 0.01828; and crude-adjusted model III: OR = 1.087 (95% CI: 1.024–1.154), *p* = 0.00599. Model IV, which has been fully modified, includes adjustments for age, gender, race, education level, marital status, PIR, BMI, smoking habit, drinking habit, hypertension, diabetes, white blood cell count, hematocrit, hemoglobin, CVD, mean corpuscular hemoglobin concentration, mean corpuscular hemoglobin, neutrophils, lymphocytes, mean corpuscular volume, red blood cell distribution width, and total platelet count. Model IV showed that a rise in MPV level was linked to greater likelihood of moderate/severe periodontitis, with an OR (odds ratio) = 1.090 (95% CI: 1.019–1.166, *p* = 0.01211). This suggests that with every incremental rise in MPV level, there is a corresponding 9.0% increase in the probability of having moderate/severe periodontitis.

To ensure the robustness of the results, continuous independent variables were converted to categorical variables. The stratification of MPV levels resulted in the creation of three groups of categorical variables and P for trend was shown in Table 2. The calculated risk rate for T2 and T3 MPV levels in the fully adjusted model was 3.0% and 21.6%, respectively, when compared to the reference T1. The P-value for the trend was 0.00617.

**Fig. 2:**
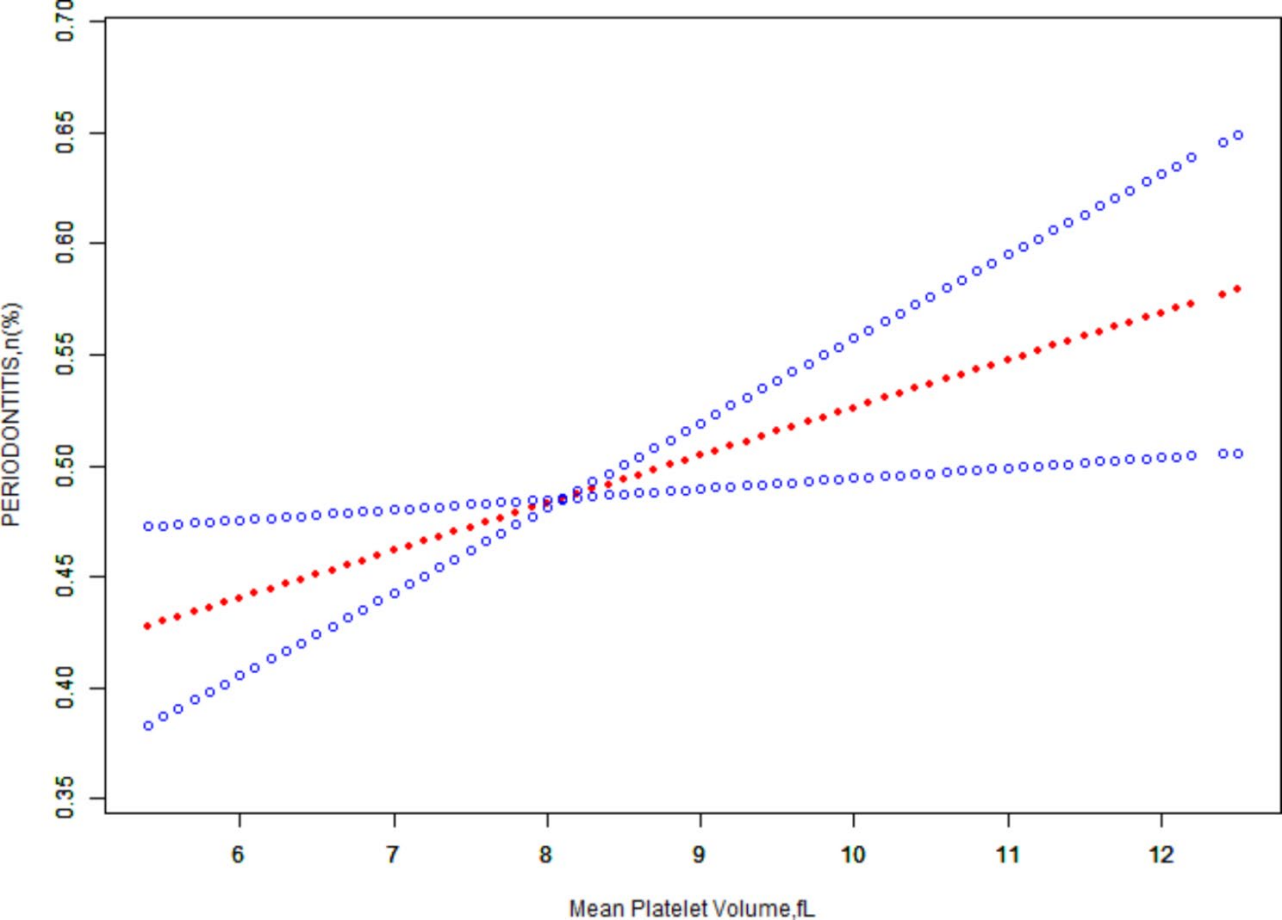
A linear association between mean platelet volume and periodontitis. Red line shows a fitted smooth curve that depicts the relationship between mean platelet volume and periodontitis while the blue bands reflect the 95% of confidence interval derived from the fit. Adjusted for age, gender, race, marital status, education level, PIR, BMI, smoking habit, drinking habit, white blood cell count, hypertension, diabetes, cardiovascular disease, mean corpuscular volume, hemoglobin, red blood cell distribution width, hematocrit, mean corpuscular hemoglobin concentration, neutrophils, lymphocytes, mean corpuscular hemoglobin, total platelet count

To further clarify the relationship between MPV and periodontitis, we performed smoothed curve fitting between MPV levels and periodontitis. The plot of adjusted smoothed curve fit, based on Fig. 2, showed a positive linear relationship between MPV levels and periodontitis.

**Table 2:** Association between Mean Platelet Volume and periodontitis in different models

Exposure	Model I	Model II	Model III	Adjust IV
	(OR,95%CI, P-value)	(OR,95%CI, P-value)	(OR,95%CI, P-value)	(OR,95%CI, P-value)
MPV (f L)	1.068 (1.015, 1.123) 0.01193	1.072 (1.012, 1.137) 0.01828	1.087 (1.024, 1.154) 0.00599	1.090 (1.019, 1.166) 0.01211
**MPV categories** **(f L)**				
T1(5.4–7.6)	1.0	1.0	1.0	1.0
T2 (7.7–8.3)	1.072 (0.950, 1.210) 0.25769	1.031 (0.899, 1.183) 0.65939	1.030 (0.896, 1.184) 0.67956	1.030 (0.894, 1.187) 0.68117
T3 (8.4–12.5)	1.187 (1.060, 1.330) 0.00296	1.182 (1.039, 1.345) 0.01131	1.212 (1.061, 1.383) 0.00448	1.216 (1.052, 1.406) 0.00816
p-value for trend	0.00272	0.00878	0.00321	0.00617

Model I adjust for: none

Model II adjust for: age; race; gender; education level; marital status; and PIR

Model III adjust for: age; gender; race; education level; marital status; PIR; BMI; smoking habit; drinking habit; hypertension; diabetes; and CVD

Model IV adjust for: age; gender; race; education level; marital status; PIR; BMI; smoking habit; drinking habit; hypertension; diabetes; CVD; mean corpuscular volume; hemoglobin; hematocrit; mean corpuscular hemoglobin; white blood cell count; neutrophils; red blood cell distribution width; mean corpuscular hemoglobin concentration; lymphocytes; and total platelet count

MPV, mean platelet volume; PIR, ratio of family income to poverty; BMI, body mass index; CVD, cardiovascular disease; OR, odds ratio; CI, confidence interval

### Subgroup analysis

To assess the consistency in association between MPV and periodontitis among population variables, we employed subgroup analysis and interaction tests. Analyses were stratified by age, gender, race, education level, marital status, PIR, BMI, smoking habit, drinking habit, and chronic diseases including (hypertension, diabetes, CVD). Table 3 shows a positive association between mean platelet volume (MPV) levels and the presence of moderate/severe periodontitis in individuals less than 40 years of age (OR = 1.19; 95% CI: 1.05, 1.34) and between the ages of 40 and 60 (OR = 1.11; 95% CI: 1.03, 1.20). A positive correlation was found between MPV and moderate/severe periodontitis in both males (OR = 1.09; 95% CI: 1.01, 1.17) and females (OR = 1.08; 95% CI: 1.01, 1.16). Furthermore, MPV was positively associated with moderate/severe periodontitis in low-income individuals (OR = 1.13; 95% CI: 1.03, 1.25), obesity (OR = 1.13; 95% CI: 1.04, 1.22), never smoke (OR = 1.07; 95% CI: 1.00, 1.15), heavy drink (OR = 1.11; 95% CI: 1.02, 1.21), had hypertension (OR = 1.07; 95% CI: 1.00, 1.15), and had no CVD (OR = 1.07; 95% CI: 1.01, 1.12). However, none of the interaction factors exhibited statistical significance (P interaction > 0.05).

**Table 3:** Subgroup analysis for the association between Mean Platelet Volume and periodontitis

Subgroup	No/Mild	Moderate/Severe OR (95%CI)	P-value	P for interaction
**Age(years)**				0.2187
< 40	Ref	1.19 (1.05, 1.34)	0.0068	
>=40, < 60	Ref	1.11 (1.03, 1.20)	0.0075	
>=60	Ref	1.04 (0.94, 1.14)	0.4664	
**Gender**				0.8798
Male	Ref	1.09 (1.01, 1.17)	0.0229	
Female	Ref	1.08 (1.01, 1.16)	0.0361	
**Marital status**				0.8701
Married/Living with a partner	Ref	1.06 (1.00, 1.13)	0.0598	
Widowed/divorced/separated	Ref	1.10 (0.99, 1.22)	0.0853	
Never married	Ref	1.06 (0.91, 1.24)	0.4525	
**Race**				0.5525
Mexican American	Ref	1.11 (0.96, 1.28)	0.175	
Non-Hispanic White	Ref	0.99 (0.91, 1.07)	0.8092	
Non-Hispanic Black	Ref	1.06 (0.94, 1.18)	0.3446	
Other	Ref	1.04 (0.93, 1.16)	0.458	
**Education level**				0.3241
High and above	Ref	1.04 (0.98, 1.10)	0.1969	
Below high school	Ref	1.11 (0.99, 1.24)	0.0704	
**PIR**				0.1684
Middle income	Ref	1.02 (0.93, 1.12)	0.6193	
Low income	Ref	1.13 (1.03, 1.25)	0.0119	
High income	Ref	1.00 (0.91, 1.10)	0.959	
**BMI**				0.0893
Not overweight	Ref	0.97 (0.88, 1.08)	0.6137	
Overweight	Ref	1.07 (0.98, 1.17)	0.1177	
Obesity	Ref	1.13 (1.04, 1.22)	0.0046	
**Smoking habit**				0.9224
Never	Ref	1.07 (1.00, 1.15)	0.0429	
Previous	Ref	1.10 (0.99, 1.22)	0.0708	
Current	Ref	1.09 (0.96, 1.23)	0.1836	
**Drinking habit**				0.2291
Never	Ref	1.08 (0.94, 1.24)	0.2822	
Moderate	Ref	1.00 (0.90, 1.09)	0.9185	
Heavy	Ref	1.11 (1.02, 1.21)	0.0139	
**Hypertension**				0.4267
No	Ref	1.03 (0.95, 1.11)	0.4547	
Yes	Ref	1.07 (1.00, 1.15)	0.0483	
**Diabetes**				0.3322
No	Ref	1.03 (0.98, 1.09)	0.2499	
Yes	Ref	1.12 (0.96, 1.30)	0.1443	
**CVD**				0.5106
No	Ref	1.07 (1.01, 1.12)	0.0196	
Yes	Ref	1.14 (0.94, 1.39)	0.1866	

PIR, ratio of family income to poverty; BMI, body mass index; CVD, cardiovascular disease

## Discussions

This study investigated the association between levels of mean platelet volume and periodontitis, as well as various factors that influence it. The study involved a total population of 3504 participants with no/mild periodontitis and 3305 participants with moderate/severe periodontitis. The results showed that U.S. adults with moderate/severe periodontitis had significant abnormalities in their blood parameters compared to those with no/mild periodontitis, mean platelet volume was positively correlated with moderate/severe periodontitis, and an increase of one unit in MPV levels was associated with a 9.0% higher probability of having moderate/severe periodontitis, and after adjusting for a wide range of potential confounders, the results of all validation analyses remained consistent and stable. Interestingly, our research revealed that participants categorized in the higher MPV tertiles were more likely to have higher odds of periodontitis. This implies that the higher the MPV, the more attention should be paid to their periodontal health. In addition, moderate/severe periodontitis showed a positive correlation with MPV, especially among participants who were age < 60, low income, obese, never smoked, heavy drinkers, had hypertension and had no cardiovascular disease.

Systemic inflammation has a significant impact on the development of periodontitis and there is evidence suggesting that periodontitis itself influences systemic inflammation [32]. However, this inflammation may be reduced with appropriate periodontal therapy. Leukocyte ratios, such as lymphocyte-monocyte ratio (LMR) and platelet-lymphocyte ratio (PLR) were suggested as potential biomarkers for systemic inflammation in the diagnosis and screening of severe periodontitis [33]. These blood parameters are simple, rapid, easy to obtain, and reflect systemic inflammation at low cost, making them promising indicators for clinical applications.

Platelets play a crucial role in the immune system [34], as they are involved in tissue damage, immunological response, and repairing processes in many diseases, in addition to their hemostatic and thrombotic effects. Recent studies have shown that MPV serves as an indicator of platelets’ involvement in inflammation and immunological response. It is also regarded as an indicator related to disease activity, inflammation and the efficacy for anti-inflammatory treatments in various inflammatory diseases, including ulcerative colitis [35], synovitis of the knee in osteoarthritis [36], and pulmonary hypertension due to chronic obstructive pulmonary disease [37].

In our study, MPV was positively correlated with moderate/severe periodontitis. This correlation may be due to the following reasons. Firstly, the inflammatory response and the progression of periodontal disease [38]. Periodontitis is essentially an inflammatory disease. Periodontal pathogens and their virulence factors activate host-associated inflammatory responses that indirectly lead to alveolar bone loss [39]. In mild periodontitis, the immune response, including the role of platelets, may be more controlled and localized and may not be sufficient to significantly alter systemic platelet levels. When periodontitis progresses to a severe phase, the systemic inflammatory response is exacerbated, which may lead to increased platelet production or activation as part of the body’s efforts to control the inflammatory state [40, 41].

Secondly, platelets’ role in inflammation. Platelets are not only involved in hemostasis but also play a crucial role in the body’s immune response. They contain and release inflammatory mediators that can exacerbate the inflammatory process [42]. Activated platelets can alter the chemotactic and adhesive functions of endothelial cells, leading to inflammation and thrombosis in the vascular system and promoting a procoagulant state of periodontitis [43]. In addition, activated platelets adhere to neutrophils via P-selectin to form complexes that work together to counteract inflammation [44]. In severe periodontitis, elevated mean platelet levels may reflect an increased systemic inflammatory response to the ongoing periodontal destruction, indicating a more aggressive or widespread inflammatory process.

Finally, the feedback mechanism. The interaction between periodontal disease and platelet activation is likely to be bidirectional [10]. Systemic inflammation caused by severe periodontitis not only activates platelets, but activated platelets also contribute to the progression of periodontal disease by promoting inflammation and potentially affecting the vascular integrity of the gingival tissues [43, 45, 46]. This feedback loop may not be as pronounced or systemic in mild periodontitis.

Nevertheless, research has shown inconsistent findings regarding the correlation between MPV and periodontitis. Some studies have revealed decreased MPV levels in patients with periodontitis patients [47], whereas other studies have shown no significant difference in MPV levels in individuals with periodontitis and those with healthy periodontal conditions [48]. This controversy may be due to the use of traditional single-center, small-sample populations to assess the relationship between periodontitis and MPV levels, which have low predictive value, as well as differences in the technical methods and measurement modalities employed. The research used data from a comprehensive nationwide survey, ensuring its reliability and dependability of the findings.

This research has several constraints. Firstly, the cross-sectional technique used in this study prevents the establishment of a causal relationship between MPV levels and periodontitis. Future prospective multicenter investigations are needed to confirm any potential causal relationships. Secondly, the MPV levels were only tested once at baseline and may not accurately reflect the prolonged state of chronic inflammation. Thirdly, these findings may not be applicable to other nationalities as they are based on US population only. Finally, because confounders were not assessed, all potential residual confounders could not be adjusted for.

## Conclusion

A positive correlation between mean platelet volume and periodontitis was confirmed in the study, and future researches are necessary to clarify the intrinsic mechanisms between them. MPV may be an important clinical marker for moderate/severe periodontitis.

## Data Availability

The data analyzed for this study can be available via download from NHANES. （https://www.cdc.gov/nchs/nhanes）.

## Abbreviations

MPV: Mean Platelet Volume
NHANES: National health and nutrition examination survey
CAL: Clinical attachment loss
PPD: Probing pocket depth
PIR: Ratio of family income to poverty
BMI: Body mass index
OR: Odds ratio
CI: Confidence interval
CVD: Cardiovascular disease
Hb: Hemoglobin
WBC: White blood cell count
Hct: Hematocrit
NEUT: Neutrophils
LYMPH: Lymphocytes
MCV: Mean corpuscular volume
MCH: Mean corpuscular hemoglobin
RDW: Red blood cell distribution width
MCHC: Mean corpuscular hemoglobin concentration
TPC: Total platelet count
